# Imbalance in the health workforce

**DOI:** 10.1186/1478-4491-2-13

**Published:** 2004-09-17

**Authors:** Pascal Zurn, Mario R Dal Poz, Barbara Stilwell, Orvill Adams

**Affiliations:** 1 Department of Human Resources for Health, World Health Organization, Geneva, Switzerland

## Abstract

Imbalance in the health workforce is a major concern in both developed and developing countries. It is a complex issue that encompasses a wide range of possible situations. This paper aims to contribute not only to a better understanding of the issues related to imbalance through a critical review of its definition and nature, but also to the development of an analytical framework. The framework emphasizes the number and types of factors affecting health workforce imbalances, and facilitates the development of policy tools and their assessment. Moreover, to facilitate comparisons between health workforce imbalances, a typology of imbalances is proposed that differentiates between profession/specialty imbalances, geographical imbalances, institutional and services imbalances and gender imbalances.

## Introduction

Imbalance in the health workforce is a major challenge for health policy-makers, since human resources – the different kinds of clinical and non-clinical staff who make each individual and public health intervention happen – are the most important of the health system's inputs [1]. Imbalance is not a new issue, as nursing shortages were reported in hospitals in the United States of America as early as 1915 [2]. It remains a major concern to this day, reported in both developed and developing countries and for most of the health care professions.

Although imbalance in the health workforce is an important issue for policy-makers, various elements contribute to obscuring policy development. First, many reports of shortages are not borne out by the evidence. Rosenfeld and Moses [3] show that an overwhelming majority of newspapers, journals and newsletter articles describing the nursing situation in the United States presume the existence of a shortage. They found that even in those areas where concrete evidence of a shortage was not available, the term "nursing shortage" still appeared. Second, the notion of shortage is a relative one: what is considered a nursing shortage in Europe would probably be viewed differently from an African perspective. Finally, imbalances are of different types and their impact on the health care system varies. In consequence, there is a general need to critically review the imbalance issue.

The objective of this paper is to contribute to a better understanding of the issues related to imbalance through a critical review of its definition and nature and the development of an analytical framework.

## Definition

There are various approaches to defining imbalances [4]. From an economic perspective, a skill imbalance (shortage/surplus) occurs when the quantity of a given skill supplied by the workforce and the quantity demanded by employers diverge at the existing market conditions [5]. Labour market supplies and demands for occupational skills fluctuate continuously, so at times there will be imbalances in the labour market. In other words, a shortage/surplus is the result of a disequilibrium between the demand and supply for labour. In contrast, non-economic definitions are usually normative, i.e. there is a shortage of labour relative to defined norms [6]. In the case of health personnel, these definitions are based either on a value judgement – for instance, how much care people should receive – or on a professional determination – such as deciding what is the appropriate number of physicians for the general population.

## Nature

One of the key questions regarding imbalances is how long these last: Is the imbalance temporary or permanent? In a competitive labour market, we should expect most imbalances to be resolved over time. Imbalances will tend to disappear faster the greater the reaction speed and also the greater the elasticity of supply (or demand) [7]. This type of imbalance (shortage or surplus) is defined as dynamic.

In contrast, a static imbalance occurs because supply does not increase or decrease; market equilibrium is therefore not achieved. For instance, wage adjustments may respond slowly to shifts in demand or supply as a result of institutional and regulatory arrangements, imperfect market competition (monopoly, monopsony) and wage-control policies. Another example is physicians' education: because of the length of time required to educate physicians, changes in available supply take a long time to react significantly. Lack of information on the state of the various labour markets can also be a factor in the speed of market adjustment. To make proper labour market decisions, households and firms must be informed of the existing market conditions across markets. They must therefore know what wages are paid and the nature and location of job openings and available workers.

Moreover, we should also differentiate between qualitative and quantitative imbalance. In a tight labour market, employers might not find the ideal candidate, but will still recruit someone. Under these conditions, the issue is the quality of job candidates rather than the quantity of people willing and able to do the job [8]. From the employers' perspective, a shortage of workers exists; from the job-market perspective, the existence of a shortage could be questioned because the jobs are filled. One negative hidden impact of a qualitative shortage is the number of positions that are filled with ineffective individuals [9].

## A conceptual framework

To better understand the role of factors affecting health workforce imbalances and to facilitate the development of policy tools, a conceptual framework is presented in this section.

### Introduction

**Figure 1:**
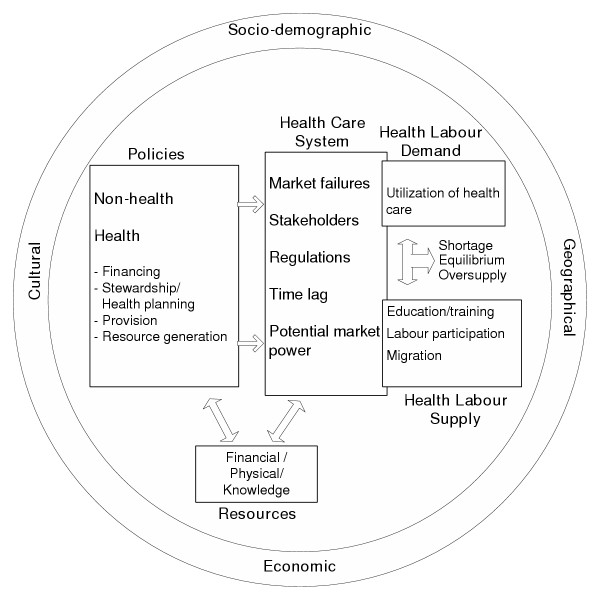
Framework for imbalance of human resources for health

Factors affecting health workforce imbalances are numerous and complex, but focusing on crucial elements should permit insight into the issue of health workforce imbalances. The framework is depicted in Fig. 1 and contains six main components: the demand for health labour, the supply of health labour, the health care system, policies, resources and "global" factors.

Central to this framework are the demand for and supply of health labour. Also included in the framework is the health care system, and in particular, some of its features that are likely to have an impact on health workforce imbalances. Policies constitute another crucial element of the framework. In effect, health policies but also non-health-oriented policies can have an impact on health workforce imbalances. The framework also incorporates financial, physical and knowledge resources that contribute to model the health workforce.Finally, "global" factors such as economic, sociodemographic, political, geographical and cultural factors are included. These elements contribute directly or indirectly to shaping and transforming the entire society and hence the health workforce.

### The demand for health personnel

The first element of the framework to be examined is the demand for health personnel. The demand for health personnel can be considered as a derived demand for health services. Accordingly, we should consider factors determining the demand for health services. Personal characteristics – such as health needs, cultural and sociodemographic characteristics – and economic factors play an important role.

It has often been proposed that the planning of human resources for health be based solely on estimates of health needs in the population [10]. However, relying only on the concept of need is difficult, because it can be defined either broadly or restrictedly and accordingly lead to a perception of either systematic shortage or surplus. Health needs is only one of the factors affecting the demand for health personnel.

Several studies have attempted to estimate the impact of economic factors on the demand for health care. In particular in the United States, studies have attempted to estimate price and income elasticities of demand for medical services [11-13]. Measurements of price or income elasticities make it possible to evaluate the impact of a change in price or income on the demand for health care. Most studies reported elasticities in the range between 0.0 and -1.0, indicating that consumers tend to be responsive to price changes but that the degree of price sensitivity is not very large compared to that for many other goods and services [14].

Another element influencing the demand for health care is the value of a patient's time, such as travel time and waiting time. Acton [15] found that in the United States, elasticity of demand with respect to travel time ranged between -0.6 and -1, meaning that a 10% increase in travel time would induce a reduction of 6%-10% in the demand for health care.

Other factors affect the demand for health labour. In particular, some specific features of the health care system and its features, policies, resources and environmental factors do have an impact on the demand for health labour. Their respective role will further discussed later.

### The supply of human resources for health

After reviewing factors affecting the demand for health labour, we shall now turn to those affecting the supply of the health workforce. In particular, we shall consider the following elements: factors affecting the choice for a health professional training/education, participation in the health labour market and migration.

### Education/professional training choice

The availability of a renewed health workforce, as well as the type of profession and specialty chosen by individuals, is a major concern for health decision-makers. These issues are of particular relevance, especially since the number of younger people, predominantly women, choosing a nursing career is declining in some countries and since in professional training/education, individuals' choices do not always match the absorptive capacity of the market.

From an economic perspective, the decision to undertake professional training/education is considered an investment decision. To emphasize the essential similarities of these investments to other kinds of investments, economists refer to them as investment in human capital [16]. Since investment decisions usually deliver payoffs over time, we must consider the entire stream of costs and benefits. The expected returns on human capital investments are a higher level of earnings, greater job satisfaction over one's lifetime and a greater appreciation of non-market activities and interests.

Based on the human capital approach, rate of return on education can be estimated. An average rate of return that is high and rising for a given profession will attract more individuals to that profession. On the other hand, a lower and decreasing average rate of return will discourage individuals from choosing that profession. Nowak and Preston [17], using the human capital approach, found that Australian nurses are poorly paid in comparison to other female professionals. The declining interest in nursing can be partly explained by the expansion of career opportunities in traditionally male-dominated occupations over the last three decades that entail a higher rate of return [18]. The number of young women entering the registered-nurse workforce has declined because many women who would have entered nursing in the past – particularly those with high academic ability – are now entering managerial and professional occupations that used to be traditionally male.

Besides the human capital approach, the choice of a profession can also be explained by sociopsychological factors. For instance, individuals may choose a profession because it is highly valued by the society or for family tradition. In the health sector, the satisfaction afforded by caring for people and assisting them to improve their health is an important element used by nursing schools to attract new enrollees. In the light of this approach, the decline in the number of individuals choosing nursing as a career might also be explained by the fact that this profession is now less socially valued than before [19,20].

### Participation in the labour market

The economic theory of the decision to work views the decision as a choice concerning how people spend their time. Individuals face a trade-off between labour and leisure. They decide how much of their time to spend working for pay or participating in leisure activities, the latter being activities that are not work-related.

An issue that has drawn a lot of attention recently is the impact of wage increases on labour participation, in particular for nurses. In the short term, higher wages can have at least two effects on the labour supply of current qualified nurses: first, qualified nurses who are working in other occupations may return to nursing activities; second, nurses now in practice may respond by working more hours. In the long run, higher wages in nursing relative to other occupations make nursing an attractive profession and will draw more people into nurse training programmes.

In their literature review of wage elasticity of nursing labour supply, Antonazzo et al. [21] and Chiha and Link [22] found that most of the studies indicate a positive relationship, although not a strong one, between wages and labour supply. Accordingly, increases in nursing wages are unlikely to cause significant increases in labour participation. A literature review on the women's workforce undertaken by Killingsworth and Heckman [23] indicated that in addition to wage rate, women's participation is responsive to changes in unearned income, spouse's wage and having children (particularly of pre-school age). Another aspect of labour supply decisions that has been investigated by Philips [24] is the costs associated with entering the nursing labour market (such as costs of child care and housework). The elasticity of participation with respect to changes in working costs was evaluated at -0.67 for all nurses. This suggests that a subsidy leading to a decrease of 10% in these costs would increase the participation of nurses by 6.7%.

Moreover, hospitals are also using a variety of strategies to recruit new staff. A survey of hospitals in the United States shows that richer benefits, such as health insurance and vacation time, are the most common incentives used. In addition, hospitals may offer other recruitment and retention benefits, such as tuition reimbursement, flexible hours and signing bonuses based on experience or length of commitment [25]. Many countries, but particularly developed ones, use such incentives to recruit new staff.

Economic factors also play a role in physician's participation in the labour market, as demonstrated by the impact of cost-containment policies in Canada, where most provincial governments have implemented an assortment of controls of health care expenses. Threshold reductions were introduced, so that fees payable to individual physicians were reduced as billing exceeded an agreed threshold. As a consequence, physicians who had billed at the threshold level chose to take leaves of absence rather than receive a level of reimbursement they considered inadequate [26].

When health personnel choose an alternative or additional occupation, this is likely to have consequences on health labour supply. In developing countries, and particularly in Africa, attempts to reform the health care sector have frequently failed to respond to the aspirations of staff concerning remuneration and working conditions. Salaries are often inadequate and may be paid late, and health workers try to solve their financial problems in a variety of ways [27]. Private practice is only one of the many survival strategies that health personnel use to supplement their income and increase their job satisfaction. Teaching, attending training courses, supervision activities, research, trade and agriculture are some of these alternative strategies [28].

### Labour market exit

Parker and Rickam [29] examined the economic determinants of the labour force withdrawal of registered nurses in the United States, i.e. nurses leaving the profession to pursue a non-nursing occupation and employed nurses withdrawing from the labour force. Their results suggest that a significant number of registered nurses withdraw, at least temporarily, from the labour force. Among the significant elements influencing the withdrawal decision are the wage rate, other family income, presence of children and full-time/part-time work status. Increasing registered nurses' wages and working full-time is expected to reduce the probability of labour force withdrawal, whereas higher education levels, age and other family income increase the probability of labour force withdrawal.

The relative importance of wage is also emphasized by studies investigating job satisfaction. There is support in the empirical literature for the existence of job dissatisfaction among nurses, and the link between job dissatisfaction and job exit [30,31]. In the United States the most important factors in nurses' resignation were, in order of importance: workload, staffing, time with patients, flexible scheduling, respect from nursing administration, increasing nursing knowledge, promotion opportunities, work stimulation, salary and decision-making. These studies suggest that salary is just one of the reasons why nurses are quitting. The relative importance of wage is confirmed by Shields and Ward [32]. Their results suggest that dissatisfaction with promotion and training opportunities has a stronger impact than workload or pay.

### Migration

Migration of health personnel can have a serious impact on the supply of human resources in health, because it may exacerbate health personnel imbalances in "sending" countries. It is suggested that migration is an "individual, spontaneous and voluntary act" that is motivated by the perceived net gain of migrating – that is, the gain will offset the tangible and intangible costs of moving [33]. Decisions to migrate are often a family strategy to produce a better income and improve survival chances [34].

The reality for many health workers in developing countries is to be underpaid, poorly motivated and increasingly dissatisfied and sceptical [35]. The relevance of motivation to migration is self-evident. There can be little doubt that for many health workers an improvement in pay and conditions will act as an incentive to stay in the country. Improved pensions, child care, educational opportunities and recognition are also known to be important [36-38]. In Ghana it is estimated that only 191 of the 489 doctors who graduated between 1985 and 1994 were still working in the country in 1997 [39].

### Health system characteristics

As the health workforce is part of the health care system, we shall also consider features of the health care system that are likely to have an impact on the demand and supply of health labour. In particular, we shall examine market failures, the diversity of stakeholders, the supply-demand adjustment time lag and hospitals' potential monopsony power.

### Market failures

From an economic perspective, the health care market is characterized by market failures – that is, the assumptions for perfect competition are violated. From a societal perspective, in the presence of market failures such as externalities – imperfect knowledge, asymmetry of information and uncertainty – market mechanisms lead to a non-optimal demand and/or supply in health services. In other words, shortages and surpluses are likely to result from the health care market.

Most markets are characterized by market failures, but what is unique to the health services market is the extent of these market failures [40]. Governments try to correct health care market failures through policy interventions. A classic example of public intervention in the presence of a positive externality is the introduction of a policy of mandatory vaccination. However, implementing such policies is sometimes difficult and may result in only partial correction of the market failures.

### Stakeholders

The health care system is characterized by a wide range of institutional stakeholders involved in shaping human resources for health, all of whom may have different objectives [41,42]. The objectives of a union or professional association do not necessarily coincide, for example, with those of a government ministry, a hospital manager or the central government. Unions/professional associations seek to increase their members' market power, employment and income [43], whereas the ministry of finance will want more budget equilibrium and will favour measures to limit health care expenditures.

In the case of Mozambique, whereas the policy of employing national professionals by cooperation agencies has met with warm support from national cadres, its effect on the health sector is problematic [44]. The prospect of immediate financial gains puts pressure on qualified professionals to leave their posts within the Mozambique National Health Service to take up management or consultant positions. The substantial investment in their training is therefore producing dubious direct returns to the National Health Service. More seriously perhaps, the presence of donor-paid jobs outside the health sector (as programme coordinators, researchers, etc.) is creating pressure on the Ministry of Health itself, exacerbating the imbalances in the National Health Service and creating incentives for trained Mozambicans to leave the public sector.

### Time lag

Moreover, adjustments between the demand and supply for health personnel may take a long time. In the health care field; the time lag between education and practising may be quite substantial. To obtain licensure to practise medicine requires lengthy education and training, and the long lag time between a changed student intake and a change in supply has been noted [45]: supply adjustment for physicians is not immediate, but takes a long time.

### Hospitals' potential monopsony power

A single entity that is the sole purchaser of labour is a *monopsony*. One example is the potential monopsony power of hospitals in hiring nurses or the ministry of health in hiring the health workforce. The amount of labour demanded will influence the price the monopsonist must pay for it. In contrast to the situation in a competitive market, the monopsony is a price maker, not a price taker. Monopsony results in lower wages and lower employment of nurses compared to a competitive market.

A number of studies have tested whether or not hospitals possess monopsony power with respect to nurses, and the results are contradictory. Sullivan [46] and Staiger et al. [47] concluded that hospitals have a substantial degree of monopsony power. In contrast, Hirsch and Schumacher [48] did not find empirical support for the monopsony model. Nurses' wages were found not to be related to hospital density and to decrease rather than increase with respect to labour market size.

### Provider power/monopoly

In contrast, providers' power may enable the latter to restrict the supply of human resources for health. Seldon, Jung and Cavazos [49] suggest that physicians in the United States have market power through such avenues as restricting supply and price-fixing. In France, trade unions are granted an institutional role at establishment level [50]. In India and Sri Lanka, a clear constraint to support-services contracting was the inability to counter the power of the public service unions in dictating employment terms and conditions [51]. The varying degree of homogeneity of the different professional groups may also explain their relative success in maintaining a monopoly of practice. In Iceland, one of the factors that contributed to breaking the professional monopoly of pharmacists was division within the profession [52].

### Regulations

The type off regulation associated with a profession plays an important role regarding the supply of members of a profession. Regulation has, by tradition, been achieved through a combination of direct government regulation and, to a large extent, through rules adopted by professional associations, whose self-regulatory powers enable them to establish both entry requirements and rules regarding professional conduct [53].

Such barriers to entry exist in particular for doctors, but also in other health professions, such as dentistry. Some argue that these barriers constitute a means to limit entry into the profession, and hence maintain high incomes. Muzondo and Pazderka [54] established, for Canadian professional licensing restrictions, a relationship between different variables of self-regulation and higher income. Seldon et al. [55] suggest that physicians in the United States have market power through such sources as restricting supply and price-fixing. However, the proponents of self-regulation claim that these barriers are a means to provide health care of quality and to protect patients from incompetent providers. In contrast, although most countries have a professional nursing association, nurses tend to have limited power to regulate entry to the profession. This could be associated with a large diversity of specialist groups in nursing failing to unite on issues related to professional regulation [56].

## Health and non-health policies

Health and non-health policies contribute to shaping the health care system and have an influence on the demand and supply of health labour.

Health policy can be defined as a formal statement or procedure within institutions (notably government) that defines priorities and the parameters for action in response to health needs, available resources and other political pressures. Health policy is often enacted through legislation or other forms of rule-making that create regulations and incentives for providing health services and programmes and access to them. For instance, the decision to introduce or expand health insurance coverage is likely to have an impact on the demand for health services. This is illustrated by the RAND Health Insurance Experiment, a controlled experiment that increased knowledge about the effect of different insurance copayments on use of medical services. Insurance copayments ranged from zero to 95%. The RAND study concluded that as the co-insurance rose, overall use and expenditure fell for adults and children combined [57].

Non-health policies reflect state interventions in areas such as employment, education and regional development that contribute to shaping the health workforce. These policies do not directly address health issues, but have an indirect impact on such issues. In France, a controversial new regulation was introduced that reduced the workweek to a maximum of 35 hours in an attempt both to create hundreds of thousands of new jobs and to achieve greater flexibility in the labour force. Unions responded by demanding the creation of more posts in public hospitals.

## Financial, physical and knowledge resources

Financial, physical and knowledge resources are crucial to any type of health care workforce. The level of resources attributed to the health care system, and how these resources are used, will have a significant impact on health workforce issues.

In terms of financial resources, human resources account for a high proportion of national budgets assigned to the health sector [58]. Health expenditure claims an increasingly important share of the gross domestic product and, in most countries, wage costs (salaries, bonuses and other payments) are estimated to account for between 65% and 80% of the renewable health system expenditure [59,60].

Physical resources include human resources within the health sector and other sectors; buildings and engineering services such as sanitation, water and heating systems for community use and for the use of medical care institutions; and equipment and supplies. Finally, the health workforce is also constrained by its human capital. This human capital can be associated with the qualification and education of the health workforce. Education of the health workforce is the systematic instruction, schooling or training given in preparation for work.

## Global factors

Economic, sociodemographic, cultural, and geographical factors contribute to shaping and transforming society and hence have a direct or indirect impact on health workforce issues.

From an economic perspective, for instance, there is evidence of a correlation between the level of economic development of a country and its level of human resources for health. Countries with higher GDP per capita are said to spend more on health care than countries with lower income, as demonstrated by cross-sectional studies, [61] and hence would also tend to have larger health workforces.

Moreover, both the demand and supply are likely to be affected by sociodemographic elements such as the age distribution of the population. On the demand side, the ageing of the population is giving rise to an increase in the demand for health services and health personnel, especially nurses for home care.

On the supply side, the ageing of the health workforce, and in particular of nurses, has serious implications for the future of the nursing labour market. For example, the Institute of Medicine noted that older registered nurses have a reduced capacity to perform certain tasks [62]. It was found that between 1983 and 1998 the average age of practising registered nurses increased by more than four years, from 37.4 to 41.9 years [63]. In contrast, the average age of the United States workforce as a whole increased by less than two years during the same period. Furthermore, the proportion of the registered-nurse workforce younger than 30 years decreased from 30.3% to 12.1% during this period.

Geographical and cultural factors also play a role in determining the demand and supply of human resources. Geographical characteristics affect the organization of health services delivery. For instance, a country with many islands or with isolated population groups will face particular challenges in terms of health workforce issues. Similarly, significant climatic changes are likely to give rise to changes in health needs, which in turn will call for changes in health services and in the health workforce. Finally, both cultural and political values also affect the demand for and supply of human resources for health.

## Health workforce imbalances: a typology

This section considers a typology of imbalances, and differentiates between the following:

• Profession/specialty imbalances: Under this category, we consider imbalance in the various health professions, such as doctors or nurses, as well as shortages within a profession, e.g. shortage of one type of specialists.

• Geographical imbalances: These are disparities between urban and rural regions and poor and rich regions.

• Institutional and services imbalances: These are differences in health workforce supply between health care facilities, as well as between services.

• Gender imbalances: These are disparities in female/male representation in the health workforce.

### Profession/specialty imbalances

Imbalances have been reported for almost all health professions, and in particular for nurses. The United States General Accounting Office [64] reports a nursing shortage. However, the nursing shortage has not been institution-wide but is concentrated in specialty care areas, particularly intensive care units and operating rooms [65]. The shortage of registered nurses in intensive care units is explained in part by the sharp decline in the number of younger registered nurses, whom intensive care units have historically attracted. Shortages in operating rooms probably reflect that many registered nurses who work in this setting are reaching the age when they are beginning to reduce their hours worked or are retiring altogether.

**Figure 2:**
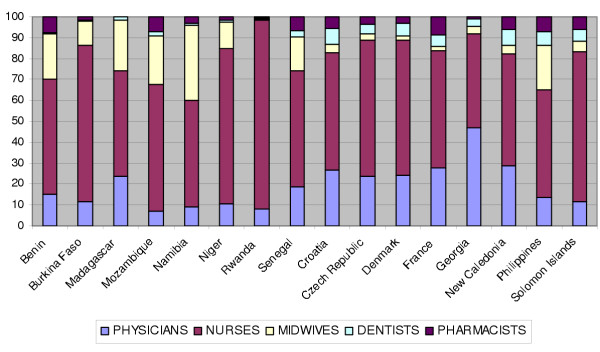
Distribution of physicians, nurses, midwives, dentists and pharmacists in selected countries. WHO data, 2000.

Major variations occur in the number of health care workers per capita population and in the skill mix employed across countries, as depicted in Fig. 2. The nurse/doctor ratio varies widely from one country to another, as shown in Fig. 2. The nurse/doctor skill mix is important and may have consequences for the respective tasks of nurses and doctors [66]. It is also interesting to note that these variations are taking place among countries with a relatively similar economic development level.

### Geographical imbalances

Virtually all countries suffer from a geographical maldistribution of human resources for health, and the primary area of concern is usually the physician workforce [67]. In both industrialized and developing countries, urban areas almost invariably have a substantially higher concentration of physicians than rural areas.

Understandably, most health care professionals prefer to settle in urban areas, which offer opportunities for professional development as well as education and other amenities for themselves and their families. But it is in the rural and remote areas, especially in the developing countries, that most severe public health problems are found.

The geographical maldistribution of doctors has been the object of particular attention. In general there is a higher concentration of general practitioners in the inner suburbs of the metropolitan areas. According to the Australian Medical Workforce Advisory Committee [68], the reasons for high concentration of general practitioners in inner city areas are:

• historical

• lifestyle-related: access to amenities

• spouse/husband-related: greater employment opportunities

• child-related: better access to secondary and tertiary education services

• professional, family and social ties and professional ambitions.

The geographical distribution of health care personnel is an important issue in many countries. Managua, the capital of Nicaragua, contains one-fifth of the country's population but around half of the available health personnel [69]. In Bangladesh, most of the doctors (35%) and nurses (30%) in health services are located in four metropolitan districts where only 14.5% of the population lives [70]. This concentration pattern is characteristic of developing countries.

In Indonesia the geographical distribution of physicians is a particular concern, since Indonesia's vast size and difficult geography present a tremendous challenge to health service delivery [71]. It is difficult to place doctors in remote islands or mountain or forest locations with few amenities, no opportunities for private practice, and poor communications with the rest of the country.

To improve the geographical distribution of physicians, governments often have used combinations of compulsory service and incentives. So far, there is virtually no country in the world that has solved the problem of a rural/urban imbalance of the physician workforce [67]. This does not necessarily mean that policies and programmes designed to reduce the imbalance have had no effect. For example, Thailand has successfully begun to stem the migration of health professionals from rural to urban areas and from public to private facilities with a range of strong financial incentives [72].

### Institutional and services imbalances

Institutional imbalances occur when some health care facilities have too many staff because of prestige, working conditions, ability to generate additional income, or other situation-specific factors, while others are understaffed [73]. Institutions such as magnet hospitals, for example, are hospitals characterized by adequate to excellent staffing, low turnover, rich nursing skill mix and greater job satisfaction, among other factors, even in the face of a general health personnel shortage [74].

Imbalance between the types of health services provided may also arise. In particular, we can consider the issue of curative versus preventive care. In effect, it has been estimated that most diseases (80%) and accidents are preventable through known methodologies, yet at present there is an imbalance in the funding of medical research, with only 1%-2% going to prevention and 98%-99% spent on curative approaches [75]. This imbalance in funding raises the question of a health workforce imbalance between preventive and curative care.

### Gender imbalances

In many countries, women still tend to concentrate in the lower-status health occupations and to be a minority among more highly trained professionals and managers. In Bangladesh, the distribution by gender of the health workforce shows that the total proportion of women accounts for little more than one-fifth in health services [76]. The distribution of women by occupational category is biased in favour of nurses. Women are very poorly represented in other categories, such as dentists, medical assistants, pharmacists, managers/trainers and doctors. The underrepresentation of women in managerial and decision-making positions may lead to less attention to and poorer understanding of the problems specific to women and the particularities of their utilization patterns [77].

Female general practitioners have been shown to practise differently from males, managing different types of medical conditions, with some differences due to patient mix and patient selectivity, and others inherent in the sex of physician. In some more traditional areas, some women will not seek care for themselves or even for their children because they do not have access to a female provider [76].

## Discussion

This framework can be used to assess policy reforms and their impact on health workforce imbalances; it also provides a common framework for cross-country comparisons. This framework emphasizes the number and type of factors affecting health workforce imbalances, illustrating the complexity of this issue. From a policy perspective, it is particularly interesting to identify factors that policy-makers can influence in order to remedy imbalance problems.

Various monetary and non-monetary incentives are used to influence the supply and/or demand for the health workforce. For example, subsidies, grants and scholarships are examples of incentives that can be used to attract more nursing students, whereas wage increases, additional benefits and working hours flexibility are examples of commonly used incentives to attract or retain the health workforce.

The numerous factors and actors involved in the health workforce imbalance issue call for a coherent health workforce vision and policy. In that context, health planning plays an important role since it contributes to shaping the health care system. Moreover, since from a societal perspective market mechanisms alone do not allow an adequate demand/supply of health personnel to be reached, public interventions such as human resources planning are a means to correct for market failures. Health planning involves a time horizon. Forecasting the future number of health personnel needed and developing policies to meet such figures are common to any health care system. Physicians represent the profession for which more planning effort has been expended to achieve a workforce of appropriate size than for any other health profession. Countries' desire to meet population health needs and to avoid social welfare losses resulting from a shortage or an oversupply are factors explaining, to a large extent, the importance attributed to planning in the context of public health policies.

The policy implications of forecasting either a shortage or a surplus of health care personnel are different, and hence attempts at projections must be rigorous. For instance, referring to previous studies predicting significant surpluses, Cooper [78] notes that such large surpluses have not occurred so far, because of a decrease in physician work effort. Factors such as age, sex and lifestyle contributed to this evolution.

As a result of forecasted physician surpluses, various policy recommendations have been formulated. The United States Institute of Medicine [79] published a report recommending, among other things, that there be no new medical schools, that existing schools should not increase their class size and that the number of first-year residency positions should be reduced. The Pew Health Professions Commission Report [80] issued a report recommending more severe steps, such as the closing of some medical schools and tightening the visa process for international medical graduates.

This framework also apprehends the different types of imbalances. This is important since the choice of a policy will also depend on the type of imbalance. Significant disparities in human resources for health between health occupation, regions, gender or health services are recognized as classic problems of imbalance. However, the question of a public/private imbalance is more debatable. One the one hand, we can argue that for equity and access, a health care system should have a strong public component. On the other hand, we can imagine a private-sector oriented health care system with mechanisms to ensure access to the poor.

## Conclusion

In an attempt to contribute to a better understanding of imbalances in the health workforce, this paper has discussed a framework for human resources for health and proposed a typology of imbalances. Although the term "imbalance" is commonly used with respect to the health workforce, it is clear that imbalance in the health workforce encompasses a wide range of possible situations and is a complex issue. The use of a framework should facilitate the development of policy tools and their assessment.

## Competing interests

None declared.

## Authors' contributions

All authors participated in writing the original text and read and approved the final manuscript.
